# Silver Nanoparticles Selectively Treat Neurofibromatosis Type 1-Associated Malignant Peripheral Nerve Sheath Tumors in a Neurofibromin-Dependent Manner

**DOI:** 10.3390/jpm12071080

**Published:** 2022-06-30

**Authors:** Garrett Alewine, Jerrica Knight, Adithya Ghantae, Christina Mamrega, Bashnona Attiah, Robert A. Coover, Cale D. Fahrenholtz

**Affiliations:** Department of Basic Pharmaceutical Sciences, Fred Wilson School of Pharmacy, High Point University, High Point, NC 27268, USA; galewine@highpoint.edu (G.A.); jknight1@highpoint.edu (J.K.); aghantae@highpoint.edu (A.G.); christinamamrega@gmail.com (C.M.); battiah@highpoint.edu (B.A.); rcoover@highpoint.edu (R.A.C.)

**Keywords:** nanomedicine, oncology, silver nanoparticles, malignant peripheral nerve sheath tumors, neurofibromatosis type 1

## Abstract

Neurofibromatosis type 1 (NF1) is among the most common neurogenic disorders, characterized by loss of function mutations in the neurofibromin gene (*NF1*). NF1 patients are extremely susceptible to developing neurofibromas, which can transform into deadly malignant peripheral nerve sheath tumors (MPNSTs). At the center of these tumors are *NF1*-null Schwann cells. Here, we found that nanomedicine shows promise in the treatment of NF1-associated MPNSTs. We assessed the cytotoxicity of silver nanoparticles (AgNPs) in *NF1*-null NF1-associated MPNSTs, *NF1*-wildtype sporadic MPNST, and normal Schwann cells. Our data show that AgNP are selectivity cytotoxic to NF1-associated MPNSTs relative to sporadic MPNST and Schwann cells. Furthermore, we found that sensitivity to AgNPs is correlated with the expression levels of functional neurofibromin. The restoration of functional neurofibromin in NF1-associated MPNSTs reduces AgNP sensitivity, and the knockdown of neurofibromin in Schwann cells increases AgNP sensitivity. This finding is unique to AgNPs, as *NF1* restoration does not alter sensitivity to standard of care chemotherapy doxorubicin in NF1-associated MPNSTs. Using an in vitro model system, we then found that AgNP can selectively eradicate NF1-associated MPNSTs in co-culture with Schwann cells at doses tolerable to normal cells. AgNP represents a novel therapy for the treatment of NF1-associated MPNSTs and addresses significant unmet clinical need.

## 1. Introduction

Neurofibromatosis type 1 (NF1) is an autosomal dominant disease that affects 1:3000, making it one of the most common neurogenic disorders [1]. NF1 is characterized by loss of function mutations in the neurofibromin gene (*NF1*), resulting in a host of comorbidities. Nearly 100% of people with NF1 will develop tumors, called neurofibromas, within the nervous system [2]. These neurofibromas can be debilitating and deadly, depending on the type and location. Unfortunately, the current standard of care for unresectable neurofibromas shows limited utility. Peripheral nerve sheath tumors (PNSTs) are a heterogeneous group of neurofibromas that affect predominantly NF1 patients. These PNSTs most often develop after a biallelic loss of functional *NF1* in the Schwann cells. PNSTs range from benign PNSTs, including plexiform neurofibromas (pNFs), to highly deadly malignant PNSTs (MPNSTs). MPNSTs are extremely aggressive soft tissue sarcomas that can develop from malignant transformation of pNFs and other atypical neurofibromas [3]. MPNSTs are the most common pediatric sarcoma and the leading cause of death in NF1 patients [4]. MPNSTs are treated with a non-specific combination of surgery, radiation, and chemotherapy and are associated with poor prognosis. Significant differences in the response to therapy exists in MPNSTs, highlighting the need to evaluate novel approaches to develop innovative treatment strategies.

Biallelic loss of functional *NF1* is the major driving force in the development of nearly all NF1-associated MPNSTs. *NF1* is a tumor suppressor gene that is universally expressed, but highly enriched in the nervous system [5]. Neurofibromin contains a RasGAP-related domain, which increases the hydrolysis rate of Ras-GTP, converting active GTP-bound Ras to the inactive GDP-bound form [6]. *NF1* is a key regulator responsible for suppressing Ras activity [5]. The role of Ras in non-cancerous tissues is to integrate external signaling to intracellular processes to promote normal cellular processes such as proliferation, survival, and differentiation through activation of downstream signaling cascades, including effectors such as PI3K, mTOR, ERK, and MAPK [7]. However these Ras-mediated pathways can be exploited during tumorigenesis. Mutations that lead to the over activation of Ras drive tumorigenesis, making it a potent oncogene [8]. Mutated and hyperactive Ras is present in nearly 33% of all cancers and is a major driving force in cancer development and progression [9]. Activating RAS mutations are not generally found in MPNSTs, rather Ras is hyper-activated due to the biallelic loss of *NF1* [10,11]. *NF1*-deficient MPNSTs require sustained Ras signaling for survival, which results in increased intracellular reactive oxygen species (ROS) [12].

Silver nanoparticles (AgNPs) are cytotoxic to a variety of cancer cells derived from an assortment of tissues at doses that have a minimal effect on normal cells [13,14,15,16,17,18,19,20,21,22,23,24,25]. We have previously optimized an AgNP that is safe for long-term repeated intravenous injection in murine models, shows no evidence of chronic toxicities, and demonstrates efficacy in preclinical models for triple negative breast cancer [25]. Mechanistically, we found that AgNPs induce DNA damage, radiosensitization, and increased protein oxidation in breast, ovarian, colorectal, prostate, and lung cancer cells [24,25,26,27]. AgNP cytotoxicity is dependent upon intracellular ROS [28], which is elevated in many cancers [12], including MPNSTs [29]. Zero-valent Ag (Ag^0^, found in AgNPs) is considered to be non-toxic. However, in the presence of hydrogen peroxide (a common intracellular ROS), the zero-valent Ag^0^ in AgNP is rapidly ionized, and the particle yielding superoxide and silver ion (Ag^+^) is degraded [28]. AgNP toxicity results from Ag^+^ release, as Ag^+^ itself is quite cytotoxic [27]. We recently illustrated the rapid degradation of AgNP in cancer cells harboring increased basal ROS using electron microscopy [25]. Corresponding imaging of AgNP-treated non-cancerous cell lines show limited AgNP degradation, demonstrating safety in non-cancerous cells. This unique mechanism of activation is the foundation for the use of AgNP as a safe cancer-selective treatment. The loss of functional *NF1* in MPNSTs causes an increase in Ras activity [6] and ROS production [7], resulting in higher basal levels of ROS relative to normal Schwann cells [29]. Therefore, we hypothesized that we could exploit this innate phenotypic difference to selectively eradicate NF1-associated MPNSTs, while leaving normal Schwann cells unharmed. In this study, we evaluated the preclinical efficacy of AgNPs as a treatment for NF1-associated MPNSTs and the role of neurofibromin expression in AgNP-mediated cytotoxicity using relevant in vitro cell models, coupling viability assays with oxidative stress inducers and gene expression alterations.

## 2. Materials and Methods

### 2.1. Cell Culture

All cells used in this study were maintained in complete culture medium (DMEM supplemented with 10% fetal bovine serum (GemCell (U.S.A. origin), GeminiBio, West Sacramento, CA, USA), penicillin (250 units mL^−1^), streptomycin (250 μg mL^−1^), and L-glutamine (2 mmol/L). Cells were used within 2 months of cryogenic resuscitation. Cells were passaged biweekly into fresh complete culture medium. ST-8814, S462TY [30], and STS26T were kindly gifted from Nancy Ratner (University of Cincinnati—Cincinnati Children’s Hospital). Immortalized Schwann cells (iHSC-1λ and iHSC-2λ) were kindly gifted from Margaret Wallace (University of Florida). The 293T cells were obtained from the American Type Culture Collection.

### 2.2. Silver Nanoparticles

Spherical silver nanoparticles stabilized by polyvinylpyrrolidone, 25 nm in diameter, were purchased from Nanocomposix, Inc., as dried powders, with a 15% silver content. Silver nanoparticles were dispersed in phosphate buffered saline (PBS), pH 7.4, without calcium or magnesium (Corning) by bath sonication. Silver nanoparticles were dispersed at a final concentration of 5 mg mL^−1^ silver content by weight, stored at 4 °C in the dark, and used within 14 days of dispersion. Silver nanoparticles were diluted directly in complete cell culture medium for viability and imaging studies. The physicochemical properties of silver nanoparticles (hydrodynamic diameter, colloidal stability, and ζ-potential) were previously characterized [24,25].

### 2.3. MTT Assays for Cytotoxicity and Viability

Cells were seeded in 96-well plates at a density of 4500–10,000 cells per well (depending on cell line and duration of experiment) in 100 μL complete growth medium (DMEM supplemented with 10% fetal bovine serum (GemCell (U.S.A. origin), GeminiBio), penicillin (250 units mL^−1^), streptomycin (250 μg mL^−1^), and L-glutamine (2 mmol/L). Cells were allowed to recover for 18 h and exposed to 2× treatment (100 μL), as indicated, for 48–72 h. To determine cell viability, 3-(4,5-Dimethylthiazol-2-yl)-2,5-diphenyltetrazolium bromide (MTT reagent (VWR); 20 μL, 5 mg mL^−1^ solution prepared in PBS), was added to each well at the appropriate time point. Plates were incubated for 45–90 min, depending on the cell line, at 37 °C/5% CO_2_. The medium was removed and replaced with 100–200 μL dimethyl sulfoxide to lyse the cells. The plates were read using a Spectramax iD5 plate reader at 560 nm and corrected for background at 650 nm, including a cell-free control well.

### 2.4. Lentivirus Production

The 293T plates were seeded in 100 mm TC dishes and allowed to attach overnight under conditions to reach ~70% confluence after 20 h. The following day, 6.4 μg envelope plasmid (psPAX (Addgene Plasmid #12259)), 1.9 μg pMD2.G (Addgene Plasmid #12260) and 5.6 μg lentiviral expression plasmids of interest—pLKO.1-puro Non-Target shRNA Control Plasmid DNA (Sigma SHC016), shNF1 (TRCN0000039713, TRCN0000039714 (Sigma)—dsRed2-RFP (Addgene 109377), or GFP (Addgene 17448) were combined in deionized water to a total volume of 100 μL in a polystyrene culture tube. Polyethylenimine (41.7 μg, linear MW 25000, Polysciences cat# 23966-1) was added to the plasmid mixture and allowed to incubate for 15 min at room temperature. The plasmid:PEI mixture was added to the 293T plates at 70% confluence in 10 mL fresh complete culture medium and incubated for 18 h at 37 °C/5% CO_2_. The following day, the medium was aspirated and replaced with 15 mL complete culture growth medium and incubated for 48 h. The medium was removed and filtered using 0.45 μM PES filter (VWR). Lentiviral supernatant was aliquoted for single-use and stored at −80 °C.

### 2.5. Neurofibromin Knockdown

Predesigned and previously validated short hairpin RNA plasmids against neurofibromin from the RNAi Consortium shRNA Library (TRCN0000039713, TRCN0000039714) and non-targeting controls were purchased from Sigma Aldrich. Lentiviral supernatants were prepared using the 293T cell line, as detailed above. STS26T, iHSC1λ, and iHSC-2λ cells (1.6–2.0 × 10^5^ cells per well) were seeded in 6-well tissue culture plates (VWR) and allowed to attach overnight. The medium was aspirated, and 0.5 mL viral supernatant (shNT—non-targeting), shNF1-713, shNF1-714, or vehicle was mixed with 0.5 mL complete growth medium and added to each well for 24 h. Cells were allowed to recover for 24 h, then medium containing puromycin (2 μg mL^−1^) was added. After selection was complete, pooled clones were propagated until a sufficient number of cells for the experiments was obtained.

### 2.6. Restoration of Functional Neurofibromin Expression

The S462TY (6 × 10^5^ cells) cells were seeded in 60 mm TC dishes and allowed to attach for 48 h. GFP expression vector (Addgene 17448) or GFP-tagged neurofibromin 1 expression vector (pLenti-NF1-GFP, kindly provided by Robert F. Hennigan (University of Cincinnati)) or vehicle (water) was added to deionized water (4.67 μg GFP plasmid; 4.67 μg for NF1-GFP-Lo; 9.33 μg for NF1-GFP-Hi) to a total volume of 100 μL. Polyethylenimine (41.7 μg, linear MW 25000) (Polysciences cat# 23966-1) was added to the plasmid mixture, mixed well, and allowed to incubate for 15 min at room temperature. The plasmid:PEI mixture was added to the S462TY cells in 3 mL fresh growth medium and incubated for 18 h at 37 °C/5% CO_2_. The following day, the medium was aspirated and replaced with 5 mL fresh growth medium and incubated for 24 h. Cells were puromycin selected (2 μg mL^−1^) until the vehicle transfected control cells were no longer viable. Cells were propagated (2 or fewer passages) until sufficient cells were available for experimental studies.

### 2.7. Realtime Quantitative PCR

Confluent plates of cells (60 mm) were harvested in 1 mL TRIZOL reagent (Invitrogen). RNA was extracted using Direct-zol RNA kits (Zymo Research), according to the manufacturer’s protocol. Quality was assessed using a DeNovix DS-11+ spectrophotometer. DNA was transcribed using 0.6–1 μg RNA input with the High Capacity cDNA Reverse Transcription Kit with RNase Inhibitor kit (Applied Biosystems) using a GeneAmp PCR System 9700 (Applied Biosystems), according to the manufacturer’s protocol, in a total reaction volume of 20 μL. Realtime quantitative PCR was performed using Taqman reagents specific for neurofibromin 1 (Hs01035108_m1) and reference gene PPIA (Hs04194521_s1) or TBP (Hs00427620_m1) [31], using 1 μL of cDNA mixture in a 384-well format in quadruplicate using Taqman Fast Advanced Master Mix (Applied Biosystems), per the manufacturer’s protocol, using a QuantStudio 6 Flex (Applied Biosystems). Relative levels of neurofibromin transcript were calculated using ∆∆Ct methodology.

### 2.8. Fluorescently Labeled Cells

S462TY (6.0 × 10^5^) or iHSC1λ (7.5 × 10^5^) cells were seeded in 60 mm TC dishes and allowed to attach overnight. The following day, the medium was aspirated and replaced with 0.5 mL viral supernatant (GFP or RFP, respectively, or vehicle, as detailed above) and 2.5 mL complete growth medium and incubated for 18 h. The following day, the medium was aspirated and replaced with 5 mL fresh growth medium and incubated for 24 h. Cells were then puromycin selected (2 μg mL^−1^) until control-transduced cells were no longer viable. GFP and RFP expression was assessed using fluorescence microscopy; cells termed S462TY-GFP and iHSC-1λ-RFP were propagated under normal growth conditions, and cryogenically preserved.

### 2.9. Other Reagents

Doxorubicin was purchased from TCI. Selumetinib was purchased from Selleck Chemical. Silver nitrate and cobalt chloride were manufactured by Acros.

### 2.10. Imaging Studies

S462TY-GFP (8.0 × 10^4^) and iHSC1λ-RFP (8.0 × 10^4^) cells were seeded in the same well in 6-well TC dishes. The following day, cells were treated with AgNP (0–50 μg mL^−1^ Ag) or vehicle in 3 mL culture medium for 0–72 h. Each day, triplicate wells were washed once in 1 mL Live Cell Imaging Solution (Gibco), and then 1 mL Live Cell Imaging Solution was added. Cells were imaged using a EVOS FL Microscope (Life Technologies) under 10× magnification. Cells were imaged using transmitted light, GFP LED cube, and TxRed LED cube to capture all S462TY-GFP and iHSC1λ-RFP cells, respectively. After imaging, cells were harvested for immunoblotting, as detailed below. Triplicate wells containing 1.6 × 10^5^ S462TY-GFP or iHSC1λ-RFP were treated with AgNP and viability assessed by MTT, as described above, to monitor the viability of the remaining cells.

### 2.11. Western Blot

Cell lysates were harvested in MPER protein extraction reagent (Thermo Scientific, Waltham, MA, USA) supplemented with Halt™ Protease Inhibitor Cocktail (Thermo Scientific). Cell lysates were sonicated briefly, and protein content was assessed by BCA protein assay (Thermo Fisher, Waltham, MA, USA) in 96-well plates, per the manufacturer’s protocol. Protein (10 μg per lane) was diluted in 4× Laemmli Buffer (Bio-Rad, Hercules, CA, USA) and heated at 95 °C for 8 min. Lysates were loaded into 15-well Mini-Protean TGX Gels (Bio-Rad) and protein ladder (Chameleon Pre-stained Duo Color Ladder, LI-COR, Lincoln, NE, USA), and electrophoresis was performed. Proteins were transferred to nitrocellulose membranes and blocked in Intercept Blocking Buffer (TBS) (LI-COR). Blots were probed with antibodies against GFP (Cell Signaling 2555), RFP (Rockland 600-401-3795), or loading control β-actin (Cell Signaling 4970) at a dilution of 1:1000 in Intercept Blocking Buffer overnight at 4 °C with gentle rocking. Membranes were than washed 3 times for 5 min in TBS, then anti-Rabbit fluorophore-conjugated secondary antibody (IRDye 800 CW Goat Anti-rabbit (926-32211), at a dilution of 1:5000 in Intercept Blocking Buffer, was added. Membranes were rocked at room temperature for 90 min in the dark, then blots washed 3 times for 5 min in TBS, as described above. Membranes were imaged using an Odyssey Clx Imaging System (LI-COR).

### 2.12. Statistical Analysis

IC_50_ was calculated by means of Graphpad Prism 9 software using a variable slope with the least squares regression method. Significance between and within groups was determined by one-way ANOVA and Student’s t-test, where appropriate. Significance is denoted as follows: *** *p* < 0.001, ** *p* < 0.01, * *p* < 0.05, n/s *p* > 0.05.

## 3. Results

### 3.1. Silver Nanoparticles Are Selectively Cytotoxic to NF1-Associated MPNSTs

**Figure 1:**
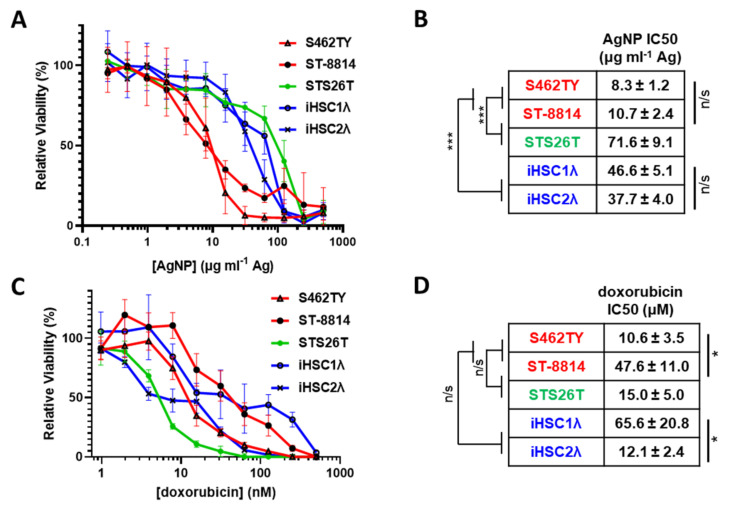
AgNPs show selective cytotoxicity against NF1-null MPNSTs relative to NF1-associated MPNSTs and normal Schwann cells, whereas standard of care doxorubicin does not show selectivity. (**A**) NF1-associated NF1-null MPNSTs (shown in red; S462TY and ST-8814), sporadic NF1-wildtype MPNST (shown in green; STS26T), and immortalized Schwann cells (shown in blue; iHSC1λ and iHSC2λ) were exposed to AgNPs (25 nm, polyvinylpyrrolidone coated, nanoComposix) for 72 h. (**B**) IC_50_ was determined for AgNP, and is shown as μg mL^−1^ Ag ± SEM. (**C**) Cells were treated with standard of care chemotherapy doxorubicin for 72 h, and viability was assessed by MTT assay. (**D**) IC_50_ of doxorubicin is shown as μM doxorubicin ± SEM. Data represent at least 4 independent experiments per cell line, each containing 5 technical replicates. Significance between and within groups was determined by one-way ANOVA and Student’s *t*-test, where appropriate (*** *p* < 0.001, * *p* < 0.05, n/s not significant).

MPNSTs most often develop in patients with NF1 and harbor a complete loss of functional neurofibromin. Rarely, MPNSTs can also develop in a sporadic manner and maintain expression of functional neurofibromin. Regardless of *NF1* mutational status, all MPNSTs share Schwann cells as the cell of origin [32]; thus, Schwann cells are an appropriate normal cell control. Therefore, to test the possibility of AgNPs as a potential therapeutic for MPNSTs, we evaluated the cytotoxicity of AgNPs in a panel of cell lines representing NF1-associated *NF1*-deficient MPNSTs (S462TY, ST-8814), sporadic *NF1*-wildtype MPNST (STS26T), and normal Schwann cells (iHSC-1λ, iHSC-2λ) as a normal cell control (Table 1). We treated our cell panel with AgNPs ranging from 0–500 μg mL^−1^ (by Ag content) for 72 h. AgNPs were highly cytotoxic to the NF1-associated (*NF1*-deficient) MPNST cell lines tested and were significantly less cytotoxic to sporadic *NF1*-wildtype MPNST and control normal Schwann cells (Figure 1A). NF1-associated *NF1*-deficient MPNSTs (shown in red) were significantly more sensitive to AgNP than normal Schwann cells (shown in blue). Sporadic MPNST (shown in green) showed a similar sensitivity to normal Schwann cells and were significantly less sensitive compared to NF1-associated MPNSTs (Figure 1B). Further, there was no intragroup difference in cytotoxicity based on IC_50_, demonstrating evidence of subtype selectivity.

The standard of care for MPNSTs of any grade is surgery, if possible, followed by doxorubicin coupled with radiotherapy. Therefore, we treated our cell panel with standard of care chemotherapy doxorubicin (0–2000 nM) for 72 h and monitored viability. In stark contrast to AgNPs, there was no clear delineation in doxorubicin sensitivity between NF1-associated MPNSTs, sporadic MPNST, and normal Schwann cells (Figure 1C). There were significant differences in doxorubicin sensitivity within groups (Figure 1D). These data suggest that there could be significant improvement in clinical outcome for NF1-associated MPNST using AgNPs compared to the standard of care doxorubicin chemotherapy.

### 3.2. Intact Silver Nanoparticles Are Required for NF1-Associated MPNST Selectivity

**Figure 2:**
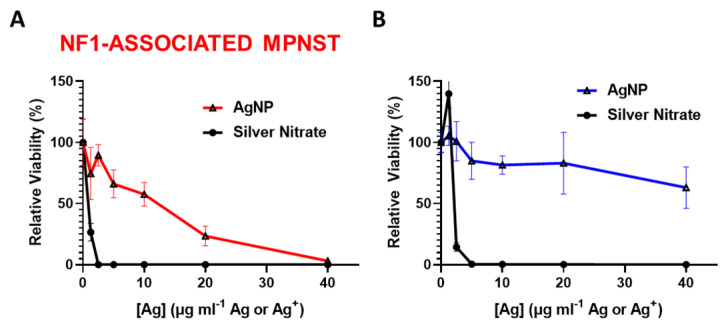
Intact AgNPs are required for NF1-associated MPNST selective cytotoxicity. (**A**) NF1-associated MPNST cells (S462TY) and (**B**) normal Schwann cells (iHSC1λ) were exposed to AgNPs (intact silver nanoparticles) or AgNO_3_ (silver ion, Ag^+^) at equivalent Ag doses for 48 h, and viability was assessed by MTT. Data are shown ± SD and are representative of 3 independent experiments containing 5 technical replicates.

We previously showed that rapid oxidation (activation) of AgNPs into silver ion (Ag^+^) in triple negative breast cancer is likely responsible for the subtype-selective AgNP-mediated cytotoxic effect [25]. To test the hypothesis that the oxidation into Ag^+^ in the cell contributes to AgNP selecticity in NF1-associated MPNST, we treated NF1-associated MPNST cells and normal Schwann cells with equivalent silver using AgNP or AgNO_3_. Silver nitrate was used as a source for Ag^+^. We found that NF1-associated MPNST (Figure 2A) and normal Schwann cells (Figure 2B) were similarly sensitive to Ag^+^, but showed differential cytotoxicity with equivalent silver mass in the form of AgNP. These data demonstrate the necessity of the nanoparticle formulation of Ag^0^ for cancer-selective therapy against NF1-associated MPNST.

### 3.3. Increased Oxidative Stress Sensitizes Normal Schwann and Sporadic MPNST Cells to Silver Nanoparticles

**Figure 3:**
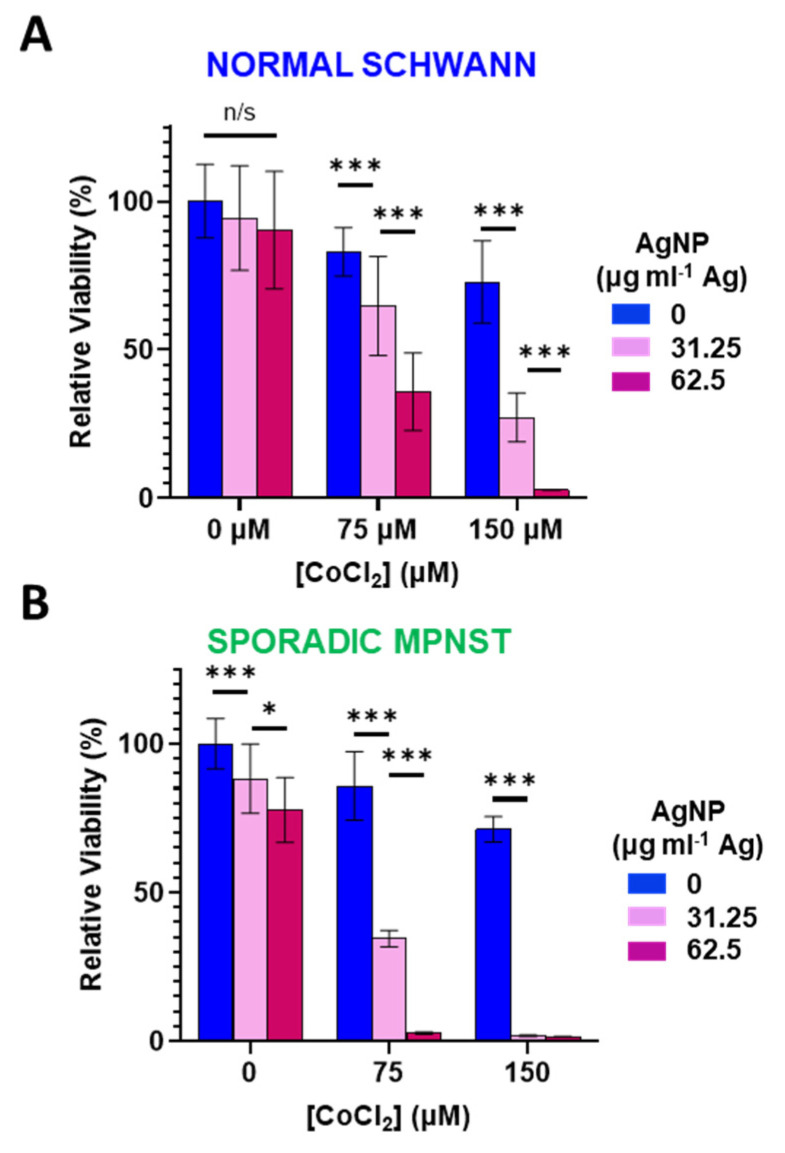
Cobalt chloride-induced hypoxic/oxidative stress sensitizes normal Schwann cells and sporadic NF1-wildtype MPNST to AgNP treatment. (**A**) Normal Schwann cells (iHSC1λ) or (**B**) sporadic NF1-wildtype MPNSTs (STS26T) were exposed to CoCl_2_ and AgNPs for 48 h, and viability was assessed by MTT. Data are shown ± SD and are representative of three independent experiments. Significance between treatments was determined by Student’s *t*-test (*** *p* < 0.005, * *p* < 0.05, n/s not significant).

Reactive oxygen species can directly degrade (and ionize) Ag^0^ from AgNPs into cytotoxic Ag^+^ [28]. Therefore, we sought to investigate the role of oxidative stress in AgNP-tolerant cells. We used cobalt chloride (CoCl_2_) as a cell tolerable inducer of oxidative stress. Mechanistically, CoCl_2_ mimics hypoxia through blockade of the Von Hippel–Lindau protein and the hypoxia-inducible factor, resulting in increased ROS and a reduction in glutathione [33,34] Normal Schwann cells (Figure 3A) or sporadic NF1-wildtype MPNST (Figure 3B) were treated with CoCl_2_ (0–150 μM) in the presence or absence of AgNP (0–62.5 μg mL^−1^ Ag) for 48 h and viability assessed. In both cell lines tested, CoCl_2_ or AgNP as single agents had only modest effects on viability. When both agents were administered simultaneously, the cytotoxicity was remarkably amplified. This evidence suggests that increasing oxidative stress potentiates AgNP-mediated cytotoxicity, and that oxidative stress may play a causal role in the mechanism of AgNP mediated cytotoxicity.

### 3.4. Restoration of Functional Neurofibromin Expression in NF1-Associated MPNST Increases Tolerance to Silver Nanoparticles

**Figure 4:**
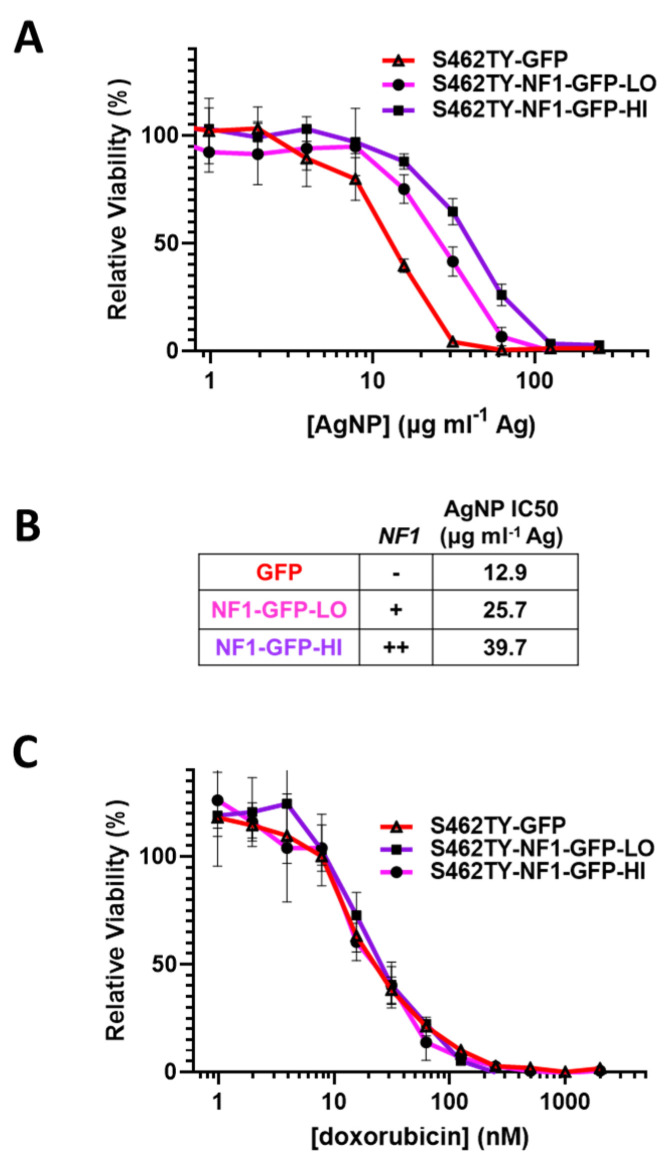
Restoration of functional neurofibromin in NF1-associated MPNST reduces sensitivity to AgNPs, with no change in sensitivity to standard of care doxorubicin. NF1-associated MPNST cell line S462TY was transfected with expression plasmids containing control GFP or two amounts of neurofibromin 1, tagged with GFP (NF1-GFP-Lo, NF1-GFP-Hi) and was puromycin selected. (**A**) Pooled cells were then treated with AgNP (0–250 μg mL^−1^ Ag) for 72 h, and viability was assessed by MTT assay. (**B**) Relative NF1-GFP plasmid levels and IC_50_ of AgNP (μg mL^−1^ Ag) are shown. (**C**) Cells were treated with doxorubicin (0–2000 nM) for 72 h, and viability was assessed by MTT assay. Data are representative of 4 independent experiments, each containing 5 technical replicates.

Sensitivity to AgNP in MPNSTs and normal Schwann cells correlates with the expression of functional neurofibromin, as we found all neurofibromin-expressing cell models are significantly less sensitive to AgNP therapy relative to cell models that lack functional neurofibromin (Figure 1A). To test whether functional neurofibromin expression had a direct effect on AgNP sensitivity, we restored neurofibromin expression in a model of NF1-associated MPNST (S462TY). Cells were transfected with two relative doses of an expression vector containing functional neurofibromin tagged with GFP (NF1-GFP), or a control expression vector containing only GFP. After puromycin selection, pooled clones of transfected cells (termed S462TY-GFP, S462TY-NF1-GFP-Lo, and S462TY-NF1-GFP-Hi) were challenged with AgNP for 72 h. Cell models with restored expression of functional neurofibromin were significantly less sensitive to AgNPs, compared to control transfected cells (Figure 4A). The *NF1* gene dose by transfected plasmid amount correlated with the IC_50_ of AgNP (Figure 4B). These data provide evidence that functional neurofibromin expression is inversely related to AgNP-mediated cytotoxicity.

It is critical to further evaluate whether this loss of therapeutic sensitivity is unique to AgNP, or rather a pan-desensitization mediated through global signaling alterations induced by the restoration of functional neurofibromin. Therefore, we treated our cell models (S462TY-GFP, S462TY-NF1-GFP-Lo, S462TY-NF1-GFP-Hi) with standard of care chemotherapy doxorubicin, as described above. All cell derivatives were equally sensitive to doxorubicin (Figure 4C). This finding is particularly intriguing, as the alterations in therapeutic sensitivity after neurofibromin restoration is unique to AgNP, and do not occur in all therapies.

### 3.5. Knockdown of Neurofibromin Sensitizes Normal Schwann Cells, but Not Sporadic MPNSTs, to Silver Nanoparticles

**Figure 5:**
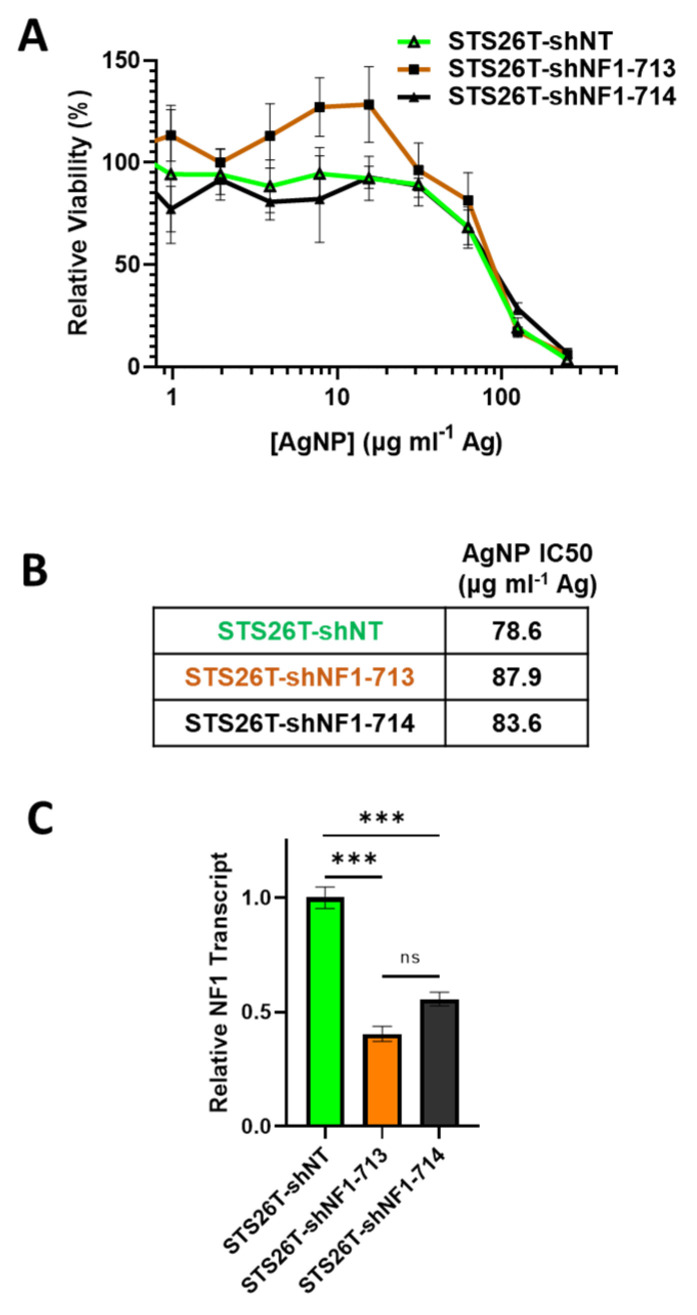
*Knockdown of neurofibromin does not alter sensitivity to AgNPs in sporadic MPNSTs*. Sporadic MPNST cells (STS26T) were transduced with non-targeting control shNT or shNF1 (713 or 714) and puromycin selected. (**A**) STS26T-shNT, -shNF1-713, and -shNF1-714 were treated with AgNP (0–250 μg mL^−1^ Ag) for 72 h, and viability was assessed by MTT assay. IC_50_ of AgNP is shown as μg mL^−1^ Ag. (**B**) AgNP IC_50_ is shown as μg mL^−1^ Ag. (**C**) qPCR specific for NF1 and the housekeeping gene TBP was performed in quadruplicate, and relative neurofibromin transcript levels ± SD calculated using ΔΔCT methods are shown. Data are representative of 3 independent experiments, each containing 4 technical replicates. *** *p* < 0.001, n/s *p* > 0.05.

Sporadic MPNSTs arise from a distinct mechanism compared to NF1-associated MPNST and often maintain expression of functional neurofibromin [3]. We next sought to test if we could sensitize *NF1*-wildtype cells to AgNPs by reducing neurofibromin expression. Thus, sporadic MPNST (STS26T) cells were transduced with neurofibromin-targeting validated short hairpin RNA (shRNA) lentiviral constructs (shNF1-713 and shNF1-714), or non-targeting controls (shNT), sourced from the publicly available RNAi Consortium. Transduced cells were selected with puromycin, and pooled clones were treated with AgNP for 72 h. We found that knockdown of neurofibromin does not alter sensitivity to AgNP in sporadic MPNST (Figure 5A,B). Knockdown was verified by quantitative PCR (Figure 5C). This provides evidence that AgNP would be the most useful in NF1-associated MPNSTs, and likely not effective in sporadic MPNSTs.

**Figure 6:**
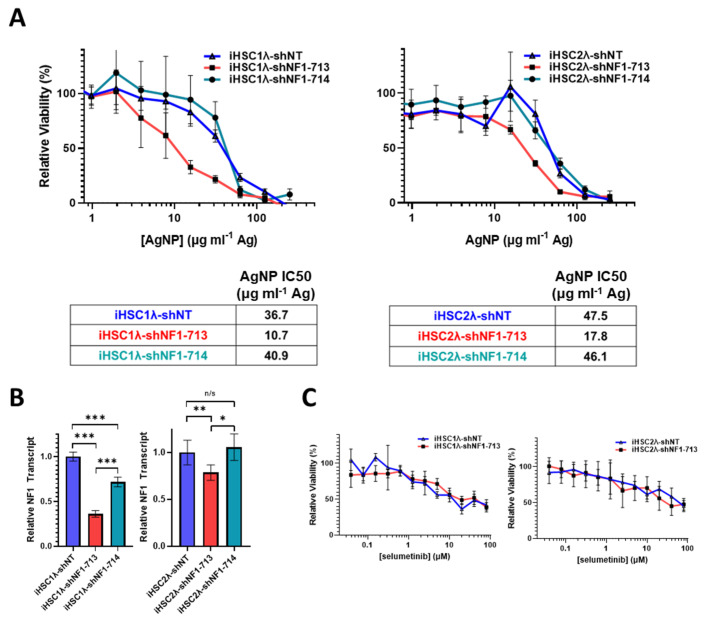
Knockdown of neurofibromin increases sensitivity to AgNPs in normal Schwann cells, with no change in sensitivity to plexiform neurofibroma standard of care selumetinib. Normal Schwann cell lines iHSC1λ and iHSC2λ were transfected with non-targeting control shNT or shNF1 (713 or 714) and puromycin selected. (**A**) iHSC1λ-shNT, -shNF1-713, and -shNF1-714 (left) or iHSC2λ-shNT, -shNF1-713, and -shNF1-714 (right) were treated with AgNP (0–500 μg mL^−1^ Ag) for 72 h, and viability was assessed by MTT assay. IC_50_ of AgNP is shown as μg mL^−1^ Ag. (**B**) qPCR specific for NF1 and the housekeeping gene PPIA was performed in quadruplicate, and relative NF1 transcript levels ± SD were calculated using ΔΔCT methods, as shown. (**C**) iHSC1λ-shNT and -shNF1-713 (left) or iHSC2λ-shNT, -shNF1-713 (right) were treated with standard of care selumetinib for 72 h, and viability was assessed by MTT. Data are representative of 3 independent experiments, each containing at least 4 technical replicates. *** *p* < 0.001, ** *p* < 0.01, * *p* < 0.05, n/s *p* > 0.05.

In NF1 patients, peripheral nerve sheath tumors develop after the complete loss of functional *NF1* in normal Schwann cells. Based on the finding that the restoration of functional *NF1* decreases sensitivity to AgNPs, we sought to investigate whether the reduction of neurofibromin expression would increase sensitivity to AgNPs in tumor cells of the Schwann origin cells. Knockdown of neurofibromin expression was performed, as described above, in normal Schwann cell lines (iHSC1λ, iHSC2λ). Knockdown of neurofibromin increased sensitivity to AgNP in both normal Schwann cell lines tested in a gene dose dependent manner (Figure 6A). Neurofibromin knockdown was validated using quantitative PCR (Figure 6B). Since loss of functional *NF1* in normal Schwann cells is an initiating event in the formation of plexiform neurofibroma, and ultimately, in malignant transformation, we challenged the neurofibromin-reduced and control cells with standard of care MEK inhibitor selumetinib. We found that there were no alterations with sensitivity to selumetinib, regardless of neurofibromin expression (Figure 6C). Similar to previous data, neurofibromin-mediated alterations in therapeutic sensitivity are unique to AgNP. Our evidence again suggests that AgNP sensitivity correlates with the expression of neurofibromin in a dose-dependent manner in NF1-related MPNSTs and cell models of cancer initiation.

### 3.6. Silver Nanoparticles Selectively Remove NF1-Associated MPNST Cells in Co-Culture with Normal Schwann Cells

**Figure 7:**
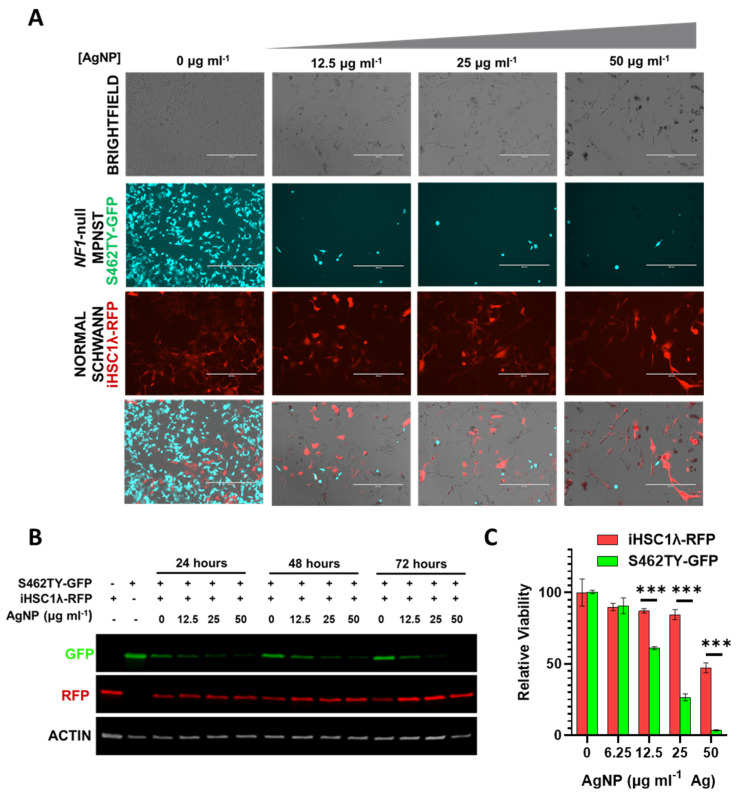
AgNP selectively removes NF1-associated MPNSTs from a co-culture with normal human Schwann cells. NF1-associated MPNST S462TY cells were transfected with a GFP expression vector, normal Schwann cell line iHSCλ1 were transfected with a RFP expression vector, and stable pooled clones were propagated after puromycin selection. iHSC1λ-RFP and S462TY-GFP were seeded at a 1:1 ratio and treated with AgNP (0–50 μg mL^−1^ Ag) for 0–72 h. (**A**) Representative images are shown after 72-h treatment with AgNP. (**B**) Cells treated as indicated were lysed and the protein was subjected to immunoblot assay for GFP, RFP, and actin control. (**C**) iHSC1λ-RFP and S462TY-GFP monocultures were treated with AgNP for 72 h, and viability was assessed by MTT. *** *p* < 0.001.

The tumor microenvironment contains multitudes of cell types comprising normal cells and cancer cells. Successful treatment of cancer in the clinic ultimately relies on the ability to safely eradicate cancerous tissue while leaving the normal tissues unharmed. Our findings show that there is likely a therapeutic window separating NF1-associated MPNSTs and normal Schwann cells. In order to model whether our AgNPs would be able to safely remove cancerous cells while leaving normal cells unharmed, we employed a co-culture system. In order to track individual cell types, we stably transduced NF1-associated MPNST cells to express green fluorescent protein (termed S462TY-GFP) and normal Schwann cells to express red fluorescent protein (termed iHSC1λ-RFP). An equal number of cells were seeded in the same culture dish and then treated with increasing concentrations of AgNP. Microscopy showed that AgNP could eradicate NF1-associated MPNST S462TY-GFP cells, while allowing normal Schwann cell iHSC1λ-RFP to remain (Figure 7A). Relative levels of RFP and GFP were also visualized by immunoblot assay (Figure 7B). To ensure that iHSC1λ-RFP were indeed viable at the same concentrations, monocultures of iHSC1λ-RFP and S462TY-GFP were treated with AgNP at the same concentrations as the co-culture, and viability was assessed by MTT (Figure 7C). This model system demonstrates that a therapeutic window may exist for the safe eradication of MPNST, while leaving normal Schwann cells unharmed.

## 4. Discussion

MPNSTs are the most common pediatric sarcoma and the leading cause of death in NF1 patients [4]. The current standard of care consists of surgery, when possible, chemotherapy (doxorubicin), and radiation therapy. The five-year survival rate of NF1-associated MPNST patients is a discouraging 52.5% [35]. Improvements must be made in the clinical management of MPNSTs, as tumors are often unresectable and respond poorly to chemotherapy, and radiation is dangerous and contributes to additional tumor formation. Rationally designed and administered nanomedicine, in this case AgNPs, provides a novel avenue for therapy and addresses significant unmet clinical need. We found that NF1-associated MPNSTs are sensitive to AgNPs in a neurofibromin-dependent mechanism at doses that are tolerable to normal Schwann cells.

Silver nanoparticles (AgNPs) are cytotoxic to a variety of cancer cells derived from an assortment of tissues at doses that have a minimal effect on normal cells [13,14,15,16,17,18,19,20,21,22,23,24,25,36]. To the best of the authors’ knowledge, this report is the first use of AgNP as a novel therapy for the treatment of MPNSTs. AgNPs show a pleiotropic mechanism of action with a variety of cytotoxic effects. Of note, AgNPs are known to induce protein oxidation [27], DNA damage in the form of double-strand breaks [24,27], misfolded proteins [37], mitochondrial dysfunction [38], redox state imbalance [24,38], endoplasmic reticulum stress [25], and lipid peroxidation [36]. In general, AgNPs induce catastrophic cell injury in specific subsets of cancer cells, resulting in both apoptotic and necrotic death in breast and lung cancers. It is likely that AgNPs induce damages similar to those observed in other cancers, and subsequent apoptosis and necrosis in NF1-associated MPNSTs [25,37]. Across tumors of disparate origins, one major commonality correlated with AgNP sensitivity is the mesenchymal-like status of the cells. Expression of mesenchymal-like markers (ZEB1, CDH1) is correlated with AgNP sensitivity in breast [25,27,36], lung [38,39], and ovarian [24] cancer cells. NF1-associated MPNSTs are also enriched in mesenchymal-like features relative to normal Schwann cells [40], and our data supports a strong correlation between mesenchymal-like status and inherent susceptibility to AgNP therapy.

Selectivity is paramount to the clinical translation of novel therapeutics. We found that NF1-associated MPNSTs are ~4.4-fold more sensitive to AgNP relative to normal Schwann cells, showing that there may be a therapeutic window for the clinical use of AgNP. This therapeutic window is unprecedented, compared to standard of care chemotherapy doxorubicin, as we found that there was no clear trend in doxorubicin sensitivity between cancer and non-cancer in the case of MPNSTs and normal Schwann cells (Figure 1). While these studies are limited to in vitro cell models, our data support that AgNP is more selective compared to the current standard of care, and further preclinical studies involving relevant in vivo model systems are warranted.

One major concern with novel therapy is safety during translation from bench to bedside. In preclinical studies using murine models, we found that systemically administered AgNPs (3 times per week, 10 weeks of treatment, 6 mg kg^−1^ Ag, through intravenous injection) were well tolerated and showed efficacy against triple-negative breast cancer [25]. No major untoward side effects were noted in rodents during the study. Importantly, IC_50_ between NF1-associated MPNST cells (S462TY) and breast cancer cells used in vitro and in orthotopic tumors [25] (MDAMB231) were quite similar (11.9 ± 7.8 and 8.3 ± 1.2 μg mL^−1^ Ag, respectively). This provides further evidence that sufficient doses of AgNP could be systemically administered to safely treat NF1-associated MPNST using in vivo models. AgNPs also show no cytotoxic effect on normal cells derived from liver, kidney, and monocyte lineages at cancer therapeutic doses [27]. A recent case report shows the efficacy of homemade AgNPs against metastatic head and neck carcinoma of the nasal cavity [41]. Self-administered AgNP treatment was initiated after multiple rounds of standard treatment regimens had failed. Remarkably, no toxicities were observed and a complete radiographic resolution of cancer was achieved. The patient was found to be cancer-free at follow up 18 months later [41]. Our in vitro data, coupled with clinical case reports, provide strong justification for the further evaluation of AgNPs for treatment of NF1-associated MPNSTs using preclinical in vivo model systems.

While our studies show strong evidence for safety, no therapeutic is without risk. To minimize untoward effects, it may be possible to use expression levels of functional neurofibromin as a biomarker for treatment with AgNP to ensure we are only treating patients that would benefit. This would be prudent, based on our findings that AgNP may not be useful in the treatment of sporadic MPNST, as they were significantly less sensitive than NF1-associated MPNST. This stark contrast may have to do with the dramatic differences in the etiology of NF1-associated MPNST compared to sporadic MPNST, as each arises via distinct mechanisms [42]. Firstly, not all sporadic MPNSTs (including the STS26T model used in this study) harbor a loss of functional *NF1* and PRC2 components, which are seen in the majority of NF1-associated MPNSTs [43,44]. Further evaluation of these mediators and studies with additional models of sporadic MPNST would be useful. However, it is clear that neurofibromin-regulated pathways which alter sensitivity to AgNP therapy in NF1-associated MPNSTs and the tumor cell of origin Schwann cells exist.

An important finding in this study is that a reduction in neurofibromin expression is sufficient to sensitize normal Schwann cells to AgNPs (Figure 6A). Loss of neurofibromin 1 is a ‘first hit’ and a major driving force in the development of atypical neurofibromas, plexiform neurofibromas, and ultimately, MPNSTs in NF1 patients [45]. Our data demonstrates that AgNP may be able to selectively remove pre-cancerous cells from NF1 patients at an extremely early stage. There may be a possible use of AgNP as a preventative therapy in NF1 patients who are prone to develop MPNSTs. This would be a major advancement in the clinical management of NF1 patients and must be investigated further.

## 5. Conclusions

In summary, we found that NF1-associated MPNSTs are exquisitely sensitive to AgNPs, compared to normal Schwann cells and sporadic MPNSTs. We then found that AgNP-mediated cytotoxicity can be influenced with alterations in basal oxidative stress. Further investigations showed that AgNP-mediated cytotoxicity is dependent on the expression of neurofibromin, as the restoration of functional neurofibromin in NF1-associated MPNST cells decreased the sensitivity to AgNP, and the knockdown of neurofibromin increased the AgNP-mediated cytotoxicity of normal Schwann cells. These data represent a unique approach for a new therapeutic strategy in the clinical management of NF1-associated MPNSTs and show that further preclinical investigations are justified.

## Figures and Tables

**Table 1:** In vitro cell models are annotated by *NF1* status and classification.

Cell Line	*NF1* Status	Subtype Classification
**S462TY**	**−/−**	**NF1-associated MPNST**
**ST-8814**	**−/−**	**NF1-associated MPNST**
**STS26T**	**+/+**	**Sporadic MPNST**
**iHSC1λ**	**+/+**	**Normal Schwann Cell**
**iHSC2λ**	**+/+**	**Normal Schwann Cell**

Red is NF1 associated MPNST, green is sporadic MPNST, and blue is normal scwhann cell in entire paper.

## Data Availability

The datasets presented in this study are available upon request from the corresponding author.
